# Expansion and Contraction of the Indo-Pacific Tropical Rain Belt over the Last Three Millennia

**DOI:** 10.1038/srep34485

**Published:** 2016-09-29

**Authors:** Rhawn F. Denniston, Caroline C. Ummenhofer, Alan D. Wanamaker, Matthew S. Lachniet, Gabriele Villarini, Yemane Asmerom, Victor J. Polyak, Kristian J. Passaro, John Cugley, David Woods, William F. Humphreys

**Affiliations:** 1 Department of Geology, Cornell College, Mount Vernon, IA 52314, USA; 2 Department of Physical Oceanography, Woods Hole Oceanographic Institution, Woods Hole, MA 02543, USA; 3 Department of Geological and Atmospheric Sciences, Iowa State University, Ames, IA 50011 USA; 4 Department of Geoscience, University of Nevada Las Vegas, Las Vegas, NV 89154, USA; 5 IIHR-Hydroscience and Engineering, University of Iowa, Iowa City, IA 52240, USA; 6 Department of Earth and Planetary Sciences, University of New Mexico, Albuquerque, NM 87131, USA; 7 Australian Speleological Federation, Perth, Western Australia, Australia; 8 Department of Parks and Wildlife, Broome, Western Australia, Australia; 9 School of Earth and Environment Sciences, University of Adelaide, Adelaide, South Australia

## Abstract

The seasonal north-south migration of the intertropical convergence zone (ITCZ) defines the tropical rain belt (TRB), a region of enormous terrestrial and marine biodiversity and home to 40% of people on Earth. The TRB is dynamic and has been shown to shift south as a coherent system during periods of Northern Hemisphere cooling. However, recent studies of Indo-Pacific hydroclimate suggest that during the Little Ice Age (LIA; AD 1400–1850), the TRB in this region contracted rather than being displaced uniformly southward. This behaviour is not well understood, particularly during climatic fluctuations less pronounced than those of the LIA, the largest centennial-scale cool period of the last millennium. Here we show that the Indo-Pacific TRB expanded and contracted numerous times over multi-decadal to centennial scales during the last 3,000 yr. By integrating precisely-dated stalagmite records of tropical hydroclimate from southern China with a newly enhanced stalagmite time series from northern Australia, our study reveals a previously unidentified coherence between the austral and boreal summer monsoon. State-of-the-art climate model simulations of the last millennium suggest these are linked to changes in the structure of the regional manifestation of the atmosphere’s meridional circulation.

The ITCZ represents the region of intense rainfall associated with the rising (wet) branch of the atmospheric meridional overturning circulation (including the Hadley circulation), a primary driver of energy and moisture exchange between the tropics and higher latitudes^1^,^2^ (Fig. 1). Both the formation and migration of the ITCZ represent a response to an energy imbalance across Earth’s thermal equator; on average, the ITCZ shifts seasonally between 9°N-2°S, with this latitudinal asymmetry due to the greater landmass in the Northern Hemisphere (NH) and the influence of the Atlantic meridional overturning circulation (AMOC)^2^. Positioning of the ITCZ is dynamic and varies over time in response to forcings that alter the interhemispheric temperature gradient. For example, during the last glacial period, cooling in the high northern latitudes displaced the TRB to the south, resulting in a precipitation dipole (an anti-phasing of rainfall) between monsoonal regions in the NH with those in the Southern Hemisphere (SH)^3^,^4^,^5^,^6^,^7^,^8^. During the relatively stable climatic conditions of the Holocene (the last 11,700 yr), the position and structure of the TRB has continued to change, albeit less markedly. Proxy and model-based studies have linked a slow southward movement of the TRB to changes in Earth’s orbital precession that moved NH summer toward aphelion^6^,^9^,^10^, while more punctuated southward shifts have occurred in response to high northern latitude cooling and expanded ice cover^11^,^12^,^13^, slowing of AMOC^2^,^14^,^15^, weakened solar irradiance^14^,^15^, and increased volcanic and anthropogenic aerosols^16^,^17^. Moreover, Pacific segments of the ITCZ have been shown to be highly sensitive to greenhouse gas forcing^18^, and thus understanding the nature and origins of past ITCZ and TRB variability represents a critical step toward developing accurate and holistic frameworks of global climate change and associated environmental responses.

While several studies have identified anti-phasing of monsoon rainfall between the hemispheres for the Holocene (i.e., drier in the NH/wetter in the SH during periods of reduced NH temperature^10^,^19^,^20^), a smaller number has suggested that instead of simply shifting meridionally as a coherent system, the Holocene TRB expanded and contracted in some regions, most notably Africa^21^,^22^ and the Indo-Pacific^23^,^24^. Expansion and contraction is notable in that it produces symmetrical interhemispheric changes in rainfall (i.e., drier in both the NH and SH during periods of reduced NH temperature) rather than an anti-phased response.

Fully understanding the dynamics of the Indo-Pacific TRB requires integrating high-resolution and precisely-dated reconstructions of low latitude hydroclimate spanning the past several millennia from both the austral and boreal summer ITCZ regions (the northern and southern margins of the TRB). However, the majority of paleomonsoon reconstructions have been developed in the NH tropics, while the southern margins of the TRB remain less understood. In recent years, stalagmites have become widely used paleomonsoon proxies because of their high temporal resolution, chronological accuracy and precision, and sensitivity to changes in monsoon strength through amount effects on oxygen isotopes in precipitation^14^,^25^,^26^,^27^. While numerous late Holocene stalagmite records have been developed for the East Asian Summer Monsoon (EASM), fewer similarly well-resolved stalagmite records have been constructed for the southern margin of the Indo-Australian Summer Monsoon (IASM)^27^,^28^,^29^. We have increased the resolution of a previously published stalagmite record of IASM variability^27^ from cave KNI-51 (15.3°S, 128.6°E), located in the central Australian tropics, and compared it with stalagmite records from China to provide a holistic picture of TRB dynamics across this region during the late Holocene (the last 3,000 yr). These findings are coupled with cutting-edge paleoclimate modeling for the last millennium in order to better understand TRB variability.

## Speleothem Reconstructions of the Indo-Pacific TRB

**Figure 1:**
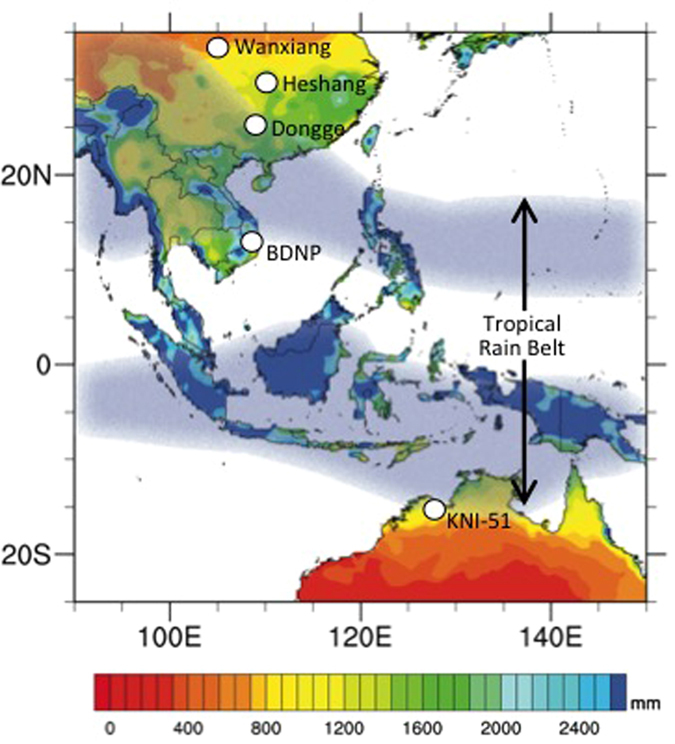
Observed mean annual rainfall (AD 1901–2010; mm/yr) for the Indo-Pacific using GPCC, v. 6^56^ with study site (KNI-51) and other cave sites discussed in text. BDNP = Bidoup Nui Ba National Park, Vietnam, site of tree ring study^37^. Blue shaded areas represent austral and boreal summer ITCZ as defined using high reflective cloud analysis^33^. Map constructed using NCAR Command Language (NCL) version 6.2.0.

We investigated late Holocene dynamics of Indo-Pacific hydroclimate by comparing the newly enhanced oxygen isotope record from cave KNI-51 to stalagmite time series located along a north-south transect through the EASM region of China (Fig. 1): Wanxiang cave (33.3°N, 105.0°E)^30^; Heshang cave (30.5°N, 110.4°E)^31^; and Dongge cave (25.3°N, 108.1°E)^14^. The ITCZ in this region forms a broad, diffuse band driven by strong atmospheric convection, and migrates seasonally between approximately 25°N-16°S, pulled farther poleward than the global average by summer heating of the Tibetan Plateau and the Australian shield^2^,^32^,^33^. This positions the boreal and austral ITCZ close to KNI-51 and Dongge cave, making both sites particularly well situated to capture subtle modern variations in TRB width. KNI-51 lies within the IASM, and the Chinese cave sites are located within the EASM, both of which are integrated within the larger Austral-Asian monsoon regime.

A full Holocene stalagmite record from KNI-51 was previously published^27^ using a combination of calcite stalagmites with low temporal resolution spanning the early and middle Holocene and a suite of aragonite stalagmites with moderation temporal resolution spanning primarily the late Holocene. Here we focus entirely on the late Holocene aragonite stalagmites, the resolution for which has been improved to ~4 yr, consistent with those of the Chinese cave records. The chronologies of KNI-51 stalagmites are precisely determined using ^230^Th mass spectrometry methods; errors on these dates range from ±1–30 yr (2 SD) in most cases^27^,^34^. The KNI-51 time series is constructed from nine aragonite stalagmites spanning the majority of the last 3,000 yr, with the longest single gap spanning AD 1640–1750. The oxygen isotopic compositions of KNI-51 stalagmites are similar in both values and trends with those of coeval samples, thereby suggesting an unadulterated paleohydrologic signal. The one exception is stalagmite KNI-51-10 which is characterized by δ^18^O values that are offset from coeval stalagmites by ~1‰. The origin of this discrepancy is unclear, and may be related to offsets affecting dripwater chemistry in tropical settings^35^. However, given that the isotopic trends of this sample are consistent with those in both coeval KNI-51 and Chinese stalagmites, we have included KNI-51-10 in our composite record (see ref. 27 for further discussion). However, removing this stalagmite from the KNI-51 time series does not appreciably diminish the results of this study.

## Late Holocene Variability of the Indo-Pacific TRB

**Figure 2:**
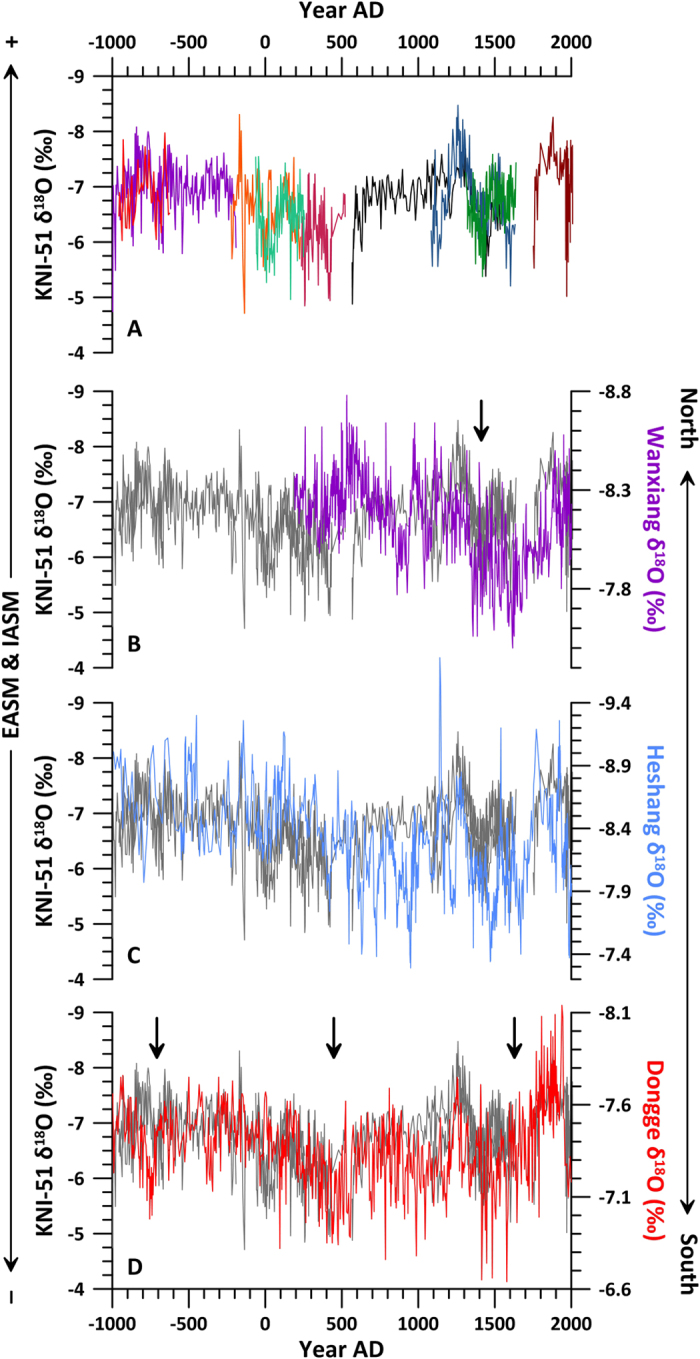
Western Pacific stalagmite monsoon records. EASM = East Asian Summer Monsoon; IASM = Indo-Australian Summer Monsoon. (**A**) Newly enhanced KNI-51 stalagmite oxygen isotope record; each stalagmite shown in a different colour. Isotope values for stalagmite KNI-51-10 (black) are shifted by 1‰^27^. (**B**–**E**) KNI-51 time series (dark grey) compared against N-S array of stalagmite time series from Wanxiang cave^30^, Heshang cave^31^ and Dongge cave^14^. KNI-51 data are not stacked but instead, where multiple stalagmites overlap in age, this composite time series uses only the data from the sample with the finer temporal resolution. Black arrow(s) in (**B**) denote Khmer Drought and in (**D**) Holocene Bond events.

The KNI-51 record is strikingly similar to the Chinese monsoon reconstructions over the late Holocene: increased monsoon rainfall (lower δ^18^O values) at KNI-51 coincides with increased rainfall northward across the Indo-Pacific TRB (Fig. 2). Because wholesale meridional displacement of the TRB would result in anti-phasing of rainfall between the hemispheres, the observation that past monsoon variability in Australia and China was synchronous and of the same sign is best explained by expansion and contraction of the Indo-Pacific TRB. The coherence at multi-decadal through multi-centennial time scales between these sites is apparent in the KNI-51 and Dongge cave records (three Holocene stalagmite records have been developed from Dongge Cave^14^,^15^,^36^; here we focus on stalagmite DA^14^). Without tuning either the DA or KNI-51 chronology, the two records are well correlated (r = 0.45 using a 251 yr loess filter and r = 0.37 using a 101-yr loess filter; Methods). Both sites feature sharp reductions in monsoon rainfall during the three ice-rafted debris (Bond) events of the late Holocene which are associated with reductions in AMOC (Fig. 2D)^14^. The KNI-51 time series also exhibits the expression in Australia of multi-decadal drought periods identified in Vietnamese tree rings^37^ and a Chinese stalagmite^30^, which have been related to the collapse of the “hydraulic city” of Angkor, the capital of Khmer Empire in Cambodia and the Chinese Yuan dynasty (the Khmer Drought is dated to AD 1340–1430^37^ and AD 1370–1460 at KNI-51) (Fig. 2B).

**Figure 3:**
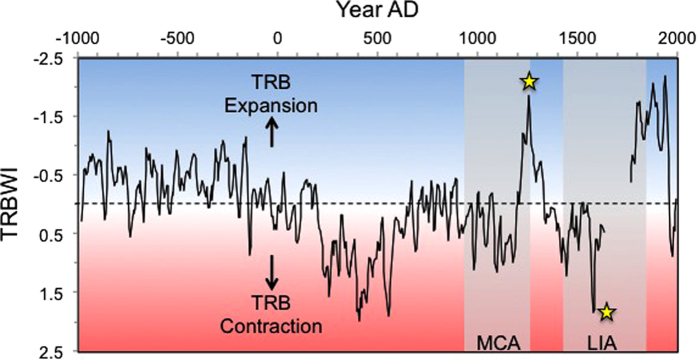
Tropical Rain Belt Width Index (TRBWI). Shown as ~15 yr running mean. TRBWI calculated as average of Z-scores of KNI-51 and Dongge cave stalagmite DA^14^ oxygen isotope values; more negative values infer strengthened average monsoon rainfall at both sites and thus a poleward migration of the austral and boreal summer ITCZ. Medieval Climate Anomaly (MCA; grey shaded region); LIA = Little Ice Age. Yellow stars denote pluvial and drought periods analysed using the Last Millennium Ensemble climate model simulations.

In order to quantify and better visualize Indo-Pacific TRB expansion and contraction, we constructed a TRB Width Index (TRBWI), calculated as the average of the Z-scores for the filtered KNI-51 and Dongge DA oxygen isotopic time series (Methods; Fig. 3). More negative TRBWI values reflect increased rainfall at both sites and thus an expanded Indo-Pacific TRB (more northerly boreal and more southerly austral summer ITCZ positions) while more positive values denote TRB contraction. Based on this evaluation, the Indo-Pacific TRB during the last millennium appears to have been narrower than during the 19^th^ and 20^th^ centuries; in contrast, one period of TRB expansion is clearly evident from AD 1200–1350. Contraction coincident with the LIA began in the Indo-Pacific at approximately AD 1400 and lasted until sometime between AD 1640–1750 (a gap in the KNI-51 stalagmite record limits our ability to precisely constrain the termination of LIA contraction). Two earlier periods of TRB contraction are also apparent: a small reduction spanning AD 900–1200, coincident with the Medieval Climate Anomaly (MCA), and a more pronounced contraction between AD 350–570 which followed the consistently expanded TRB that characterized the first millennium BC.

Expansion and contraction of portions of the global TRB have been previously identified. For example, analysis of lake sediments in Africa indicated a reduction in the width of the TRB during the LIA^21^, consistent with asymmetric contraction of African rain forest from the middle to late Holocene documented through analysis of terrestrial carbon isotopes in marine sediments^22^. In the Indo-Pacific, comparisons of carbon isotopic records of leaf waxes from Java lake sediments to paleohydrologic reconstructions from across the Indo-Pacific also revealed expansion/contraction of the regional TRB at multi-decadal to centennial time scales over the last millennium^23^. And most recently, an integrated array of paleohydrologic records from across Southeast Asia, the Maritime Continent, and Australia demonstrated contraction of the TRB during the LIA^24^. However, no previous study has so unambiguously revealed the sensitivity of TRB expansion/contraction behaviour as our integrated regional stalagmite analysis.

## Climate Model Analysis of Late Holocene Indo-Pacific TRB Dynamics

**Figure 4:**
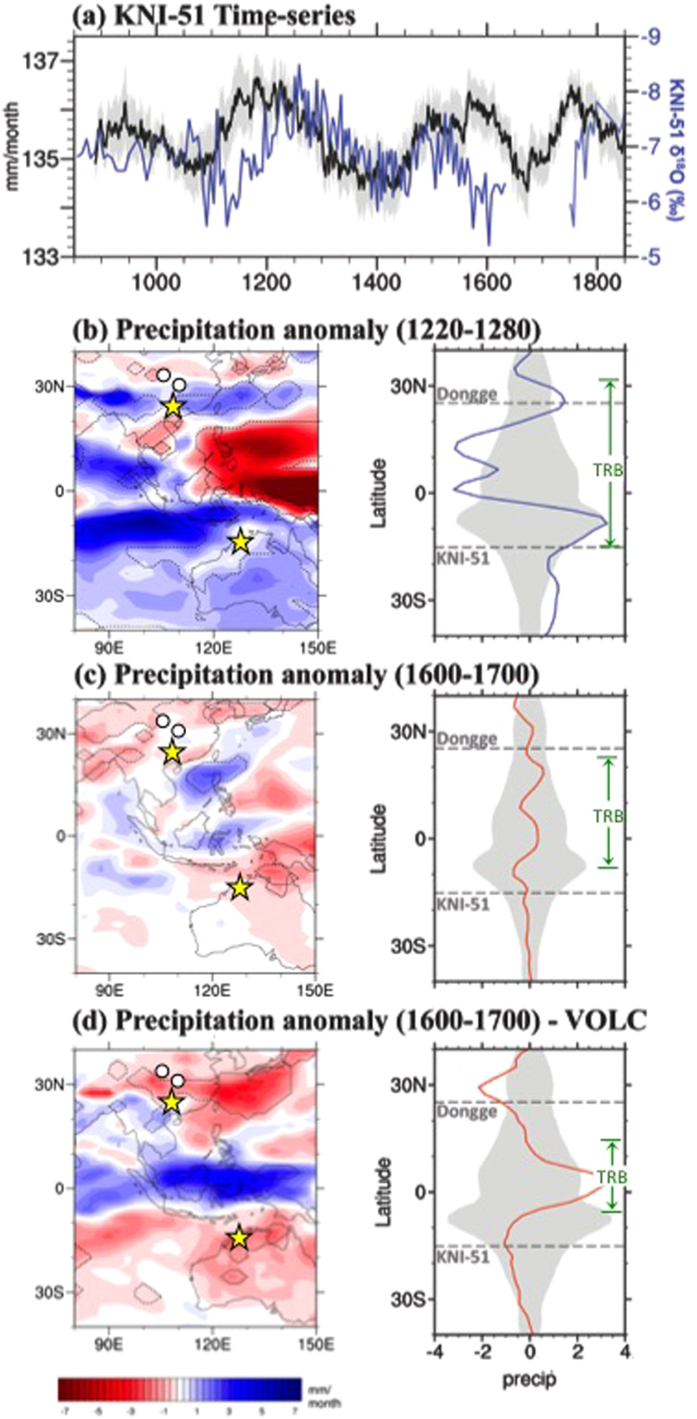
(**a**) Comparison of KNI-51 time-series from AD 850–1850 for oxygen isotope time series (blue) and simulated annual precipitation (black), with wetter conditions upward on the y-axis. The black model time series is shown as 80-yr running average and is based on the LME multi-ensemble mean with grey shading reflecting ±1 SE. Changes in monthly rainfall are averaged over the entire calendar yr and thus the range of <7 mm/month produced here equates to a change in annual rainfall of <84 mm, equal to approximately 7–10% of the mean annual rainfall at KNI-51. The apparent offsets in the two time series originate due to 50-yr offsets between modelled and reconstructed rainfall at AD 1150, 1550, and 1750. (**b**) Left: Composite map of precipitation anomaly (mm/month) during the AD 1220–1280 pluvial period, the wettest section of a longer pluvial period in both the KNI-51 and Dongge DA cave records. Dashed contours delimit areas significant at the 90% level according to a two-tailed *t*-test. Yellow stars denote locations of Dongge cave and KNI-51; white circles are the other cave sites discussed in the text (see Fig. 1). Note expansion of TRB and concomitant decrease of rainfall in the core equatorial region. Right: Precipitation anomaly during the AD 1220–1280 pluvial (blue) is zonally averaged for the Austral-Asian sector 100°–140°E. Grey shading reflects average zonal mean conditions according to Monte Carlo/boot-strapping methods. Dashed lines indicate latitude of Dongge and KNI-51 caves. Tropical Rain Belt (TRB) is denoted by width of green arrow. (**c**) Same as (**b**) but for AD 1600–1700 drought period. (**a**–**c**) Are based on the “All” forcing scenario (includes orbital, solar, volcanic, land use, greenhouse gases, and aerosols). (**d**) Same as (**c**) except based only on the volcanic forcing scenario. Note marked contraction of TRB with concomitant increase in rainfall in the core equatorial region. (**b**–**d**) Constructed using NCAR Command Language (NCL) version 6.2.0.

To examine the origins of TRB behaviour, we compared the KNI-51 and Dongge DA records from the last millennium with output from the Last Millennium Ensemble (LME)^38^, a high-resolution suite of climate model simulations conducted by the National Center for Atmospheric Research with the Community Earth System Model (Methods). The simulated precipitation over northern Australia (spanning 14°–16°S, 125°–130°E) in an area surrounding the cave site is consistent with the stalagmite monsoon reconstruction at KNI-51 over the last millennium (Fig. 4a). In addition, the model largely captures the mean climatology of NH and SH summer monsoon precipitation, though the austral monsoon precipitation extends too far into the interior of the Australian continent, a problem common to current-generation climate models^39^ (Figure S1).

To assess TRB dynamics in more detail, we focused on the most prominent multi-decadal pluvial period (AD 1220–1280) and drought period (AD 1600–1700) of the last 1,000 yr (Fig. 4; Methods). During the pluvial, composite precipitation anomalies in the LME simulations reflect wetter conditions across southern China and across northern Australia; substantial decreases in rainfall mark the central and eastern core of the TRB (Fig. 4b). This pattern is also apparent in the zonal mean plots for precipitation averaged across the Austral-Asian sector (longitude range 100°–140°E; Fig. 4b), and is consistent with an expansion of the regional TRB via a more poleward positioning of the rising branch of the tropical meridional circulation.

For the drought period, the model shows modest reductions in rainfall across the Australian tropics and southern China as well as weakly enhanced rainfall through much of the TRB core (Fig. 4c). This result is generally consistent with our stalagmite analysis as well as regional proxy-based studies for the LIA^24^. Why the model more accurately captures rainfall variability during the pluvial than the drought is unclear but may be linked to the model’s anomalously deep penetration of monsoon rainfall into the Australian interior that limits its sensitivity to changes in meridional circulation in the area of KNI-51 during TRB contractions (Fig. 1).

The LME also allows attribution of particular hydroclimatic changes to external forcing factors (Methods). Of particular interest is the role played by volcanic activity, linked to onset and enhancement of LIA cooling^40^,^41^, as well as sub-decadal droughts during the last millennium^42^. When the model is forced solely by volcanic emissions, the resulting spatial distribution of reduced monsoon rainfall during the drought period is remarkably consistent with the stalagmite data (Fig. 4d). Pronounced drought conditions occur across much of Southeast Asia and the Australian (sub)tropics, while equally dramatic increases in rainfall span the core of the TRB.

A second driver of particular relevance is solar irradiance because the three Holocene stalagmite records from Dongge cave, as well as the Wanxiang cave record^30^, contain prominent expressions of solar signals at sub- and multi-centennial scales^14^,^15^,^36^. However, despite the strong similarities between the Dongge DA and KNI-51 oxygen isotopic records, spectral characteristics suggestive of solar variability are weak or absent in the KNI-51 monsoon reconstruction (Figure S2). Whether the lack of a prominent solar signal in the KNI-51 time series is related to the assembling of multiple, short-duration stalagmite records, uncertainties in the chronologies, or a true absence of a strong solar influence on regional monsoon behaviour, is at present unclear.

Today, the two primary drivers of hydroclimate variability across the Indo-Pacific are the El Niño-Southern Oscillation (ENSO), the periodic shift in sea surface temperature (SST) and centres of atmospheric convection that represents the single largest source of inter-annual global climate variability, and the Pacific Decadal Oscillation (PDO; or Interdecadal Pacific Oscillation – IPO), a (multi)decadal-scale oscillation in (North) Pacific ocean temperature. ENSO strongly influences rainfall across Southeast Asia and many parts of Australia, although its influence in the central Australian tropics is weak^43^. The ENSO-IASM relationship is modulated by the PDO on multi-decadal timescales^44^, and the PDO has been demonstrated to produce symmetrical climatic responses in the NH and SH^45^,^46^; the PDO is suggested as a contributor to multi-decadal drought in Cambodia^37^ and Myanmar^47^. It thus appears likely that ENSO-PDO dynamics are similarly involved in Indo-Pacific TRB expansion and contraction. However, our proxy and modelling results suggest that late Holocene expansion and contraction of the Indo-Pacific TRB should not be attributed to any single climate driver. For example, the TRB contracted over centennial-scales coincident with reductions in AMOC during Holocene Bond events and also over multi-decadal periods (such as the Khmer Drought) tied to phasing of the PDO^37^, while volcanic forcing best captures the reconstructed rainfall anomalies during the LIA.

Although the influence of Indo-Pacific SST (including via ENSO) on historical monsoon variability in north-central Australia is small^43^, cooler SST during the LIA would have likely suppressed regional atmospheric moisture fluxes. Thus, the role of SST in the LME simulations must be considered when assessing the model’s ability to resolve TRB dynamics apparent in the stalagmite records. In the LME simulations, overall anomalous cool SST occurred during both the 13^th^ century pluvial and the 17^th^ century drought. The pluvial period also featured SST reminiscent of the negative phase of the PDO with localized warm SST in the western subtropical North Pacific and cool anomalies in the western/central equatorial Pacific and western Indian Ocean (Figure S3). Such a negative PDO phase SST pattern was previously associated with changes in moisture transport into Southeast Asia and anomalously wet conditions across southern China^47^, including the Dongge site, consistent with the pluvial conditions shown here. In addition, no clear sign of tropical Pacific zonal SST anomalies such as those associated with ENSO in the equatorial eastern Pacific, are manifest in the model simulations at either time (Figure S3). These results suggest that broad-scale changes to the meridional atmospheric circulation as reflected in the LME precipitation response occur through a suite of integrated forcings and represent a likely mechanism for TRB expansion and contraction.

The recognition that the TRB is capable of expansion and contraction at these time scales has important implications for our understanding of past drivers of climate variability and their feedbacks. For example, tropical wetlands in the SH represent an important source of methane^48^, particularly in areas that are seasonally flooded^49^. Thus the contribution of methane from the Australian tropics during periods of TRB expansion should be carefully considered in models of Holocene climate.

## Methods

### Site Selection

Several other stalagmite records have been developed from across Southeast Asia and the Indo-Pacific. However, we restricted our analysis to time series commensurate with that from KNI-51. For example, Gunung Buda, the site of a stalagmite paleomonsoon record in Borneo, was excluded from this analysis owing to its relatively low temporal resolution for the Holocene^5^. Liang Luar, southern Indonesia^28^,^29^, was excluded because its location on the margin of the TRB core suggests it may have experienced a more complex rainfall history associated with rain belt expansion/contraction.

### Stable Isotopic Analysis

Stable isotopic analyses of speleothem carbonate was performed at the Iowa State University (ISU) Department of Geological and Atmospheric Sciences Stable Isotope Laboratory using a Gas-Bench II with a CombiPal autosampler coupled to the inlet of a ThermoFinnigan Delta Plus XL continuous flow mass spectrometer and at the University of Nevada Las Vegas (UNLV) Isotope Science Laboratory using a ThermoElectron Delta V Plus mass spectrometer linked to a Kiel IV automated carbonate preparation device. International standards were analysed at the start and end of each run, as well as within the sample series. Analytical error is ±0.1‰ for both carbon and oxygen.

### Statistical Correlations Between Stalagmite Time Series

To test for statistical significance of the filtered correlations between Dongge and the KNI records at 5-yr interpolation time resolution, we ran a Monte Carlo simulation of 1000 random time series with the same autocorrelation structure as the Dongge record modelled as an AR1 auto-regressive process (lag-1 autocorrelation coefficient = 0.6725) with variable loess filter lengths (101 and 251-yr). The 1000 filtered random time series were correlated (r; Pearson correlation coefficient) to the 5-yr interpolated and identically-filtered KNI time series beneath the hiatus (from AD –987 to 1638), and the resulting correlation distribution was characterized by one, two, and three sigma ranges of correlations that may arise purely due to chance at the respective levels. For the 101-yr loess filter, the one, two, and three sigma correlation limits that could arise solely due to chance are r = +/−0.08, 0.16, and 0.24, respectively. For the 251-yr loess filtered data, the limits are r = +/−0.10, 0.20, and 0.29. These values compare to the observed correlations of r = 0.37 and r = 0.45 for the 101 and 251-yr loess filters, respectively. These observations suggest that the observed correlations for the 101 and 251-yr filtered time series are both significant at the three sigma (99.7%) level and did not arise either due to chance or the low-pass filters applied to the data.

### Tropical Rain Belt Width Index

The TRBWI was calculated as the average of Z-scores of the composite stalagmite δ^18^O time series using the equation Z = (x-μ)/σ where x = individual oxygen isotopic ratio, μ = mean of oxygen isotopic ratios, and σ = standard deviation of oxygen isotopic ratios. A decrease in Z-score reflects a more negative oxygen isotopic value that, through amount effects on low latitude rainfall, indicates a stronger monsoon. Amount effects in northern Australia are determined using GNIP data^50^ from Darwin, the closest station to KNI-51, and yield a correlation coefficient, r, for amount effects for September-May of −0.8 and a slope of this relationship equal to −0.9‰/100 mm/month. A detailed study of controls on oxygen isotopes in precipitation across Australia identified at Darwin^51^ the more complex relationship of δ^18^O = (6.67 × 10^−6^) P^2^ − 0.009 P + 0.015Eva + 0.007Rad − 9.670 where P is precipitation amount, Eva is evaporation, and Rad is solar radiation. For sites across southern China, an average amount of effect relationship was observed of δ^18^O = −2.27ln(P) + 3.64 (ref. 52), while near Dongge cave, amount effects are less straightforward, as evidenced by a detailed analysis of the origins of isotopic variability in precipitation at several sites as part of both GNIP and the Chinese Network of Precipitation program, CHNIP, across southern China^53^ in which a complex interaction was noted using these sites between precipitation amount, air temperature, vapour pressure, relative humidity, water pressure, sunshine duration, wind speed, and wind direction. At the two CHNIP sites closest to Dongge Cave (Huanjiang and Huitong), correlation coefficients between precipitation amount and precipitation δ^18^O values were −0.4 and −0.7. GNIP data for Guilin were analysed using a step-wise regression model and identified the following relationship: precipitation δ^18^O = 2277.891–0.015T^2^ – 758.678Vp – 1.44logP where T is temperature, Vp is vapour pressure, and P is precipitation (ref. 53).

### Climate Model Simulations

To examine the TRB expansion/contraction behaviour, a series of monthly global gridded variables from a state-of-the-art model participating in the Coupled Model Intercomparison Project, phase 5 (CMIP5) were analysed. CMIP5 represents an unprecedented archive of climate model output produced under the auspices of the Intergovernmental Panel on Climate Change (IPCC) Fifth Assessment Report (AR5). The experimental design for CMIP5 is given by ref. 54. In particular, we used output from the Last Millennium Ensemble conducted by the National Center for Atmospheric Research (NCAR) Paleoclimate group with the Community Earth System Model (CESM). The LME design followed the Paleomodel Intercomparison Project phase 3 (PMIP3)/CMIP5 protocol for the Last Millennium scenario, as detailed by ref. 55. Ensemble members extend from AD 850–1850 using reconstructions for the transient evolution of solar intensity, volcanic emissions, greenhouse gases, aerosols, land use conditions, and orbital parameters, together and individually. The LME project consists of 30 ensemble members: ten simulations with all transient forcings were used, smaller ensembles with each transient forcing separately, such as three ensemble members with the volcanic forcing only used here^38^. All model results (Fig. 4 and S2, S4) were based on the multi-ensemble member average of all 10 (for the ALL) and three (volcanic) ensemble members available for the forcing scenarios, respectively.

The pluvial and drought periods were identified as intervals of particularly pronounced and sustained hydroclimate conditions as recorded in the KNI-51 and Dongge time series. In the case of the pluvial, we selected the centre of a broad period of enhanced monsoon rainfall. For the LIA drought, the interval AD 1600–1700 was selected because the Dongge record, as well as the portion preserved in the KNI-51 record, exhibit evidence of a consistently weakened monsoon during this time. Slightly earlier intervals during the LIA exhibit a more prominent, though somewhat shorter-lived, drought signal but the model analysis focused on the most persistent drought period identified in the record.

### Data accessibility

The KNI-51 stable isotope and U-series data are included in the Supplemental Materials and are archived at the NOAA National Centers for Environmental Information website.

## Additional Information

**How to cite this article**: Denniston, R. F. *et al*. Expansion and Contraction of the Indo-Pacific Tropical Rain Belt over the Last Three Millennia. *Sci. Rep.* **6**, 34485; doi: 10.1038/srep34485 (2016).

## Supplementary Material

Supplementary Information

Supplementary Information
