# Supplementary material for: Expansion and Contraction of the Indo-Pacific Tropical Rain Belt over the Last Three Millennia
Source: Sci Rep. 2016 Sep 29;6:34485. doi: 10.1038/srep34485 (PMC5041111; doi:10.1038/srep34485)
Supplement: Supplementary Information [file srep34485-s1.pdf]

Supplemental Information

Expansion and Contraction of the Indo-Pacific Tropical Rain Belt over the Last Three Millennia

R.F. Denniston, C.C. Ummenhofer, A.D. Wanamaker, Jr., M.S. Lachniet, G. Villarini, Y. Asmerom, V.J. Polyak, K.J. Passaro, J. Cugley, D. Woods, W.F. Humphreys

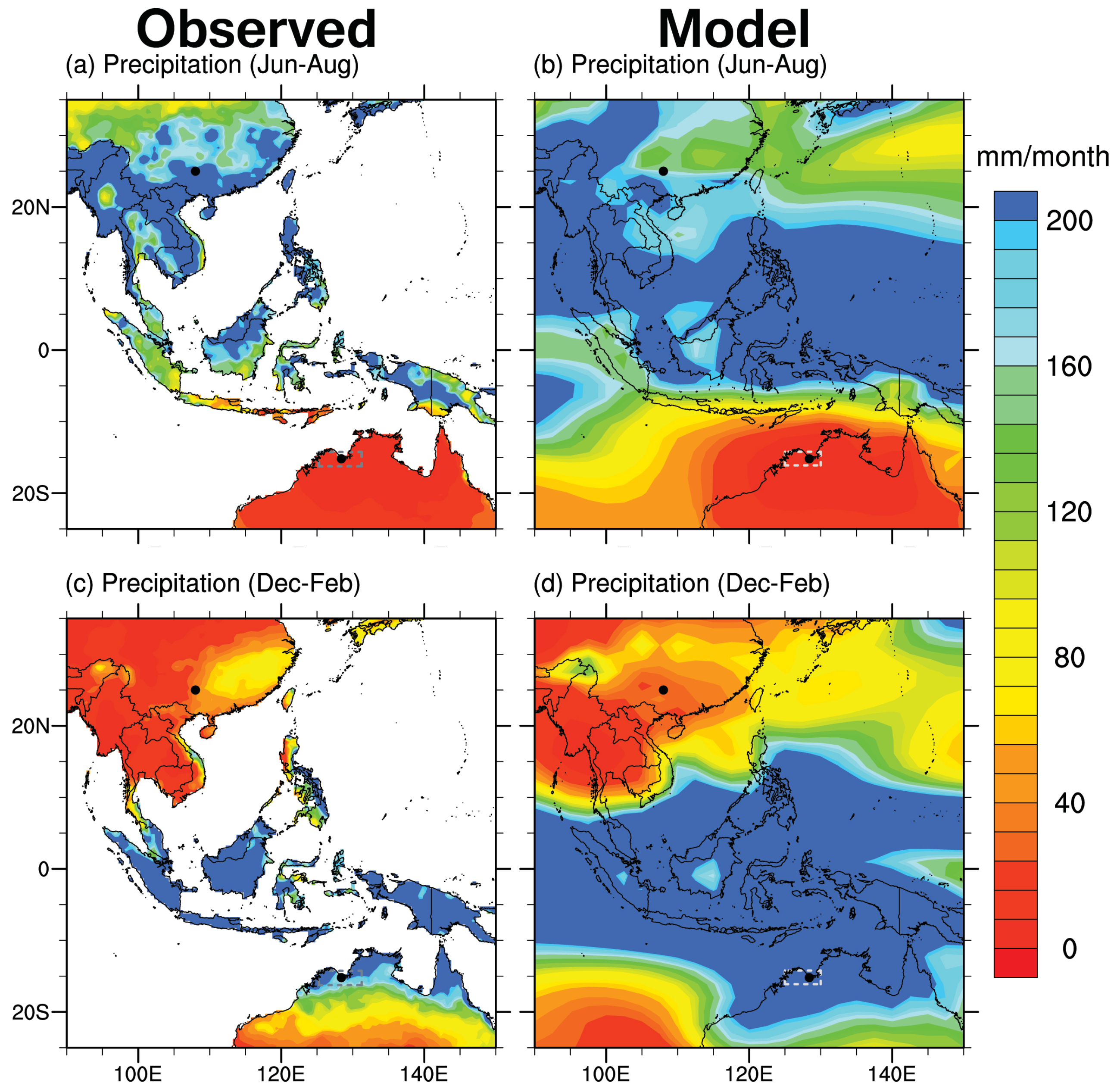

Figure S1. Mean precipitation (mm/month) using GPCC, v. 6[1] for the observations (left) and the model (right) for the June-August (JJA) and December-February (DJF) seasons for the period 1910-2004. Circles in (a-d) indicate locations of the cave sites KNI-51 and Dongge. The model climatologies are based on the multi-ensemble mean across the ALL forcing scenario. The gray boxes delimit the spatial extent in the observations and model for generating the KNI precipitation time-series, e.g., time series in Fig. 4.) Map constructed using NCAR Command Language (NCL) version 6.2.0.

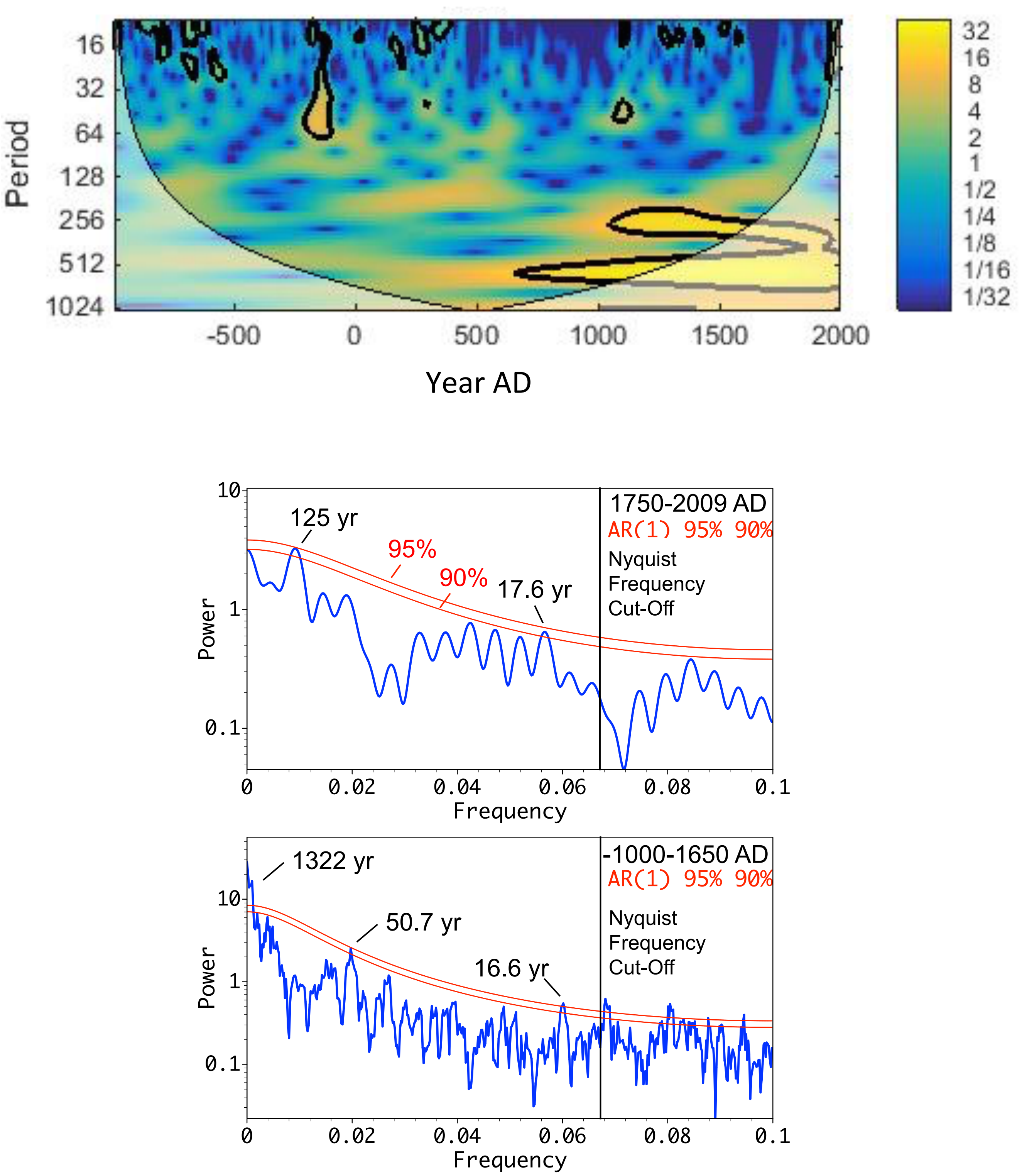

Figure S2. Spectral analysis of the KNI-51 time series using Morlet wavelet (top panel; parameter = 6, start scale = 2, scale width = 0.25, powers-of-two = 11; http://ion.exelisvis.com/)[2] and multi-taper method (bottom panel; kSpectra version 3.4.3, resolution = 2, number of tapers = 3)[3] techniques. Raw data were interpolated in 5-year bins prior to spectral analysis. Significant periods at the 95% confidence interval using an auto-regressive lag1 red noise background spectrum are shown as black contours for the wavelet analysis and above the upper most red line for multi-taper method analysis. On the wavelet plot, the shaded region is the cone of influence, where zero padding has reduced the variance[2]. Identified in the Dongge Cave record are periodicities at 558, 232, 206, 159, 148, 129, 116, 104, 89, 57, and 54 years[4] and at 340, 200, and 80 years[5].

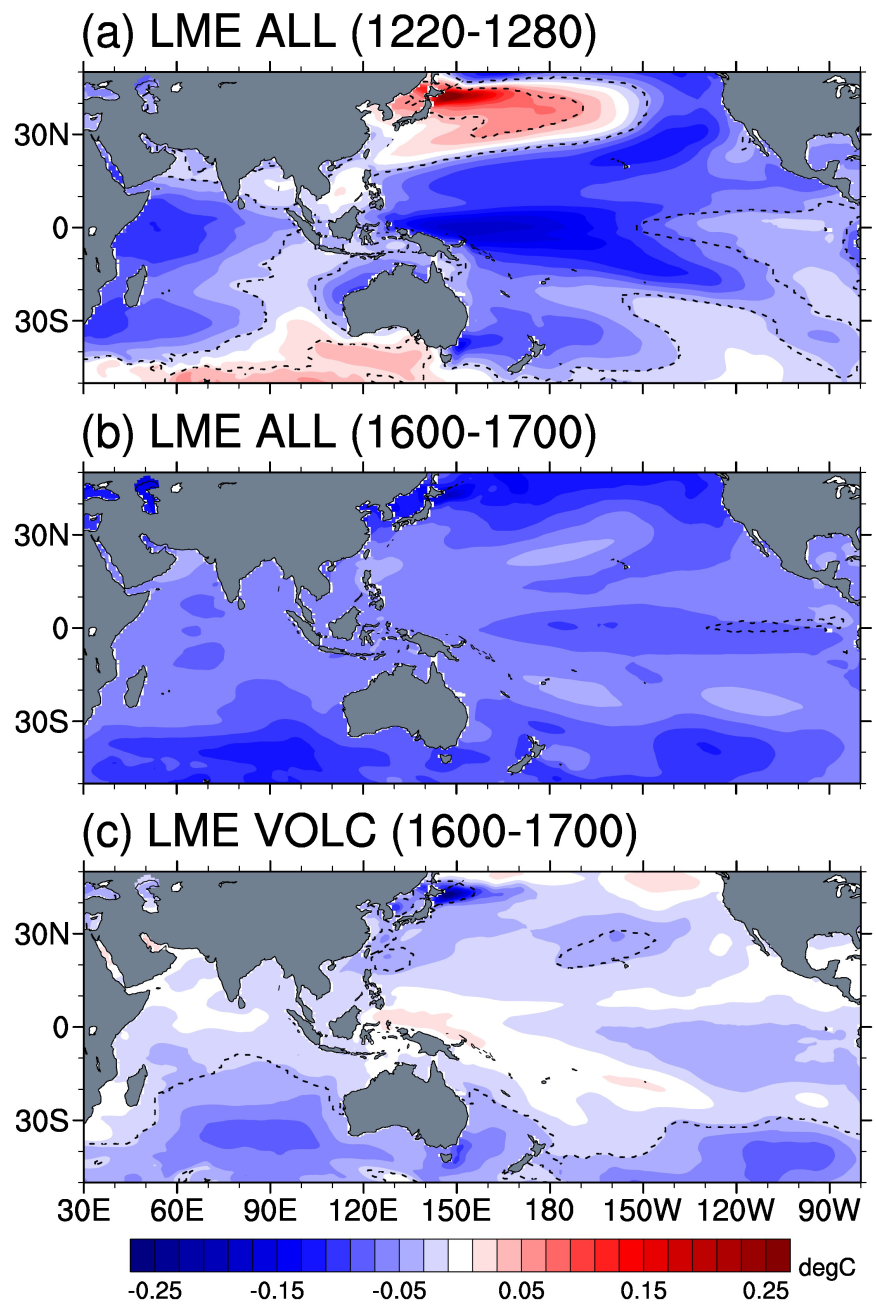

Figure S3. LME composite SST anomalies (˚C) for (a) pluvial period and (b) drought period identified in the Dongge and KNI-51 stalagmite monsoon reconstructions under the ALL forcing scenario. (c) same as (b) but for the VOLC forcing scenario. Maps constructed using NCAS Command Language (NCL) version 6.1.0-beta.

References

1. Schneider, U. *et al.* GPCC's new land surface precipitation climatology based on quality-controlled in situ data and its role in quantifying the global water cycle. *Theor App Clim* **115**, 15-40 (2013).
2. Torrence, C. & Compo, G. A practical guide to wavelet analysis. *Bull Amer Met Soc* **79**, 61-78 (1998).
3. Mann, M. & Lees, J. Robust estimation of background noise and signal detection in climatic time series. *Clim Change* **33**, 409-445 (1996).
4. Wang, Y. *et al.* The Holocene Asian monsoon: links to solar changes and North Atlantic climate: *Science* **308**, 854-857 (2005).
5. Duan, F. *et al.* Evidence for solar cycles in a late Holocene speleothem record from Dongge Cave, China. *Sci Rep* **4**, DOI: 10.1038/srep05159 (2014).
